# Retrospective Evaluation of Factors Affecting Lymph Node Retrieval Following Gastrectomies with Oncologic Intent

**DOI:** 10.5041/RMMJ.10434

**Published:** 2021-04-29

**Authors:** Steven Fuchs, Itamar Ashkenazi

**Affiliations:** 1 Surgery Department, The Brooklyn Hospital Center, New York, NY, USA; 2 The Ruth & Bruce Rappaport Faculty of Medicine, Technion–Israel Institute of Technology, Haifa, Israel; 3 Division of General Surgery, Rambam Health Care Campus, Haifa, Israel; 4 Department of General Surgery, Hillel Yaffe Medical Center, Hadera, Israel (former affiliation)

**Keywords:** Adequate resection, gastrectomy, gastric cancer, lymphadenectomy

## Abstract

**Background:**

Adequate lymphadenectomy is an important factor affecting survival in gastric cancer patients. Retrieval and examination of at least 15 lymph nodes is recommended in order to properly stage gastric malignancies. The objectives of this study were to evaluate the proportion of patients undergoing inadequate lymphadenectomies and possible risk factors for inadequate surgery.

**Methods:**

This was a retrospective study that included patients, 18 years and older, who underwent gastrectomies with oncologic intent in the Hillel Yaffe Medical Center. We analyzed the association of demographic, clinical, and pathological variables with adequate number of lymph nodes.

**Results:**

The retrieval of less than 15 lymph nodes was reported in 51% (53/104) patients undergoing gastrectomies with oncologic intent. The extent of surgery was the only variable associated with inadequate lymphadenectomy on univariate analysis: subtotal/proximal versus total gastrectomy (*P*=0.047). Differences observed for previous surgery (*P*=0.193), T stage (*P*=0.053), N stage (*P*=0.051), and lymphovascular invasion (*P*=0.14) did not reach significance. Subtotal/proximal gastrectomy resulted in inadequate resection of lymph nodes in 56% of the patients, while this occurred in only 30% of the patients undergoing total gastrectomy (relative risk 1.865; 95% CI 0.93, 3.741). Logistic regression confirmed that only subtotal/proximal versus total gastrectomy was associated with inadequate number of lymph nodes resected (*P*=0.043).

**Discussion and Conclusion:**

In this study we analyzed the association of patient, tumor, and surgery-related factors on adequate lymphadenectomy in patients undergoing gastrectomies for possible gastric cancer. Larger extent of the surgery (total, rather than subtotal/proximal gastrectomy) was revealed to be the only indicator positively associated with adequate lymphadenectomy.

## INTRODUCTION

Gastric cancer remains one of the most common cancers in the world, and the third most common cause of cancer-related mortality.^1^ Prognosis is often poor, even in developed countries, likely due to late detection and poor screening techniques. For gastric malignancies, the treatment of choice for potentially curable gastric cancers is resection, along with adjuvant or neo-adjuvant therapies depending on the level of invasiveness and metastatic potential of the cancer.^2^ The therapy is chosen based on the TNM cancer staging.^3^,^4^

**Figure 1:**
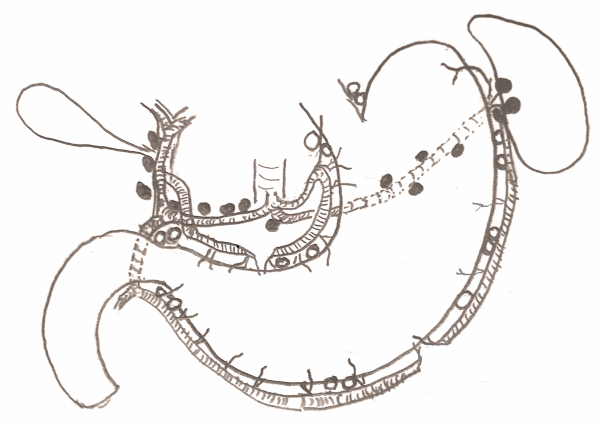
Schematic Drawing Showing Lymph Nodes Resected in D1 and D2 Gastrectomies Lymph nodes resected in D1 gastrectomies are adjacent to the stomach itself (depicted by light circles). Lymph nodes resected in D2 gastrectomies include those adjacent to the stomach as described above, and lymph nodes embedded in the fatty tissue surrounding the blood vessels arising from the celiac axis: splenic artery, common hepatic artery, and the proper hepatic artery (depicted by dark circles).

Previous studies have demonstrated that the greater the number of lymph nodes resected, the better the survival prognosis, and hence D2 was generally recommended (Figure 1).^5^ Eventually, the Dutch D1D2 trial with a 15-year follow-up results revealed that D2 resections did in fact result in lower regional reoccurrence of gastric cancer and lower mortality due to gastric cancer compared to D1 resections.^6^ Since the nomenclature of lymphadenectomy may be surgeon-related, some authors suggest that the number of lymph nodes resected is more important than their respective location.^7^ These authors suggest that pathologically examining at least 15 lymph nodes allows appropriate staging of the tumor. We therefore use the term “adequate” to describe the retrieval of 15 lymph nodes or more, and “inadequate” for the retrieval of less than 15 lymph nodes.^8^,^9^ Still, in a significant proportion of gastric resections the goal of pathologically examining at least 15 lymph nodes is not achieved.^10^

While there is an agreement as to the minimum number of lymph nodes required for appropriate assessment, only a few studies have evaluated the factors associated with achieving this goal. This study’s main objective was to examine how many surgeries indeed succeed in resecting an adequate number of lymph nodes and to evaluate possible factors associated with inadequate lymph node resection.

## MATERIALS AND METHODS

### Study Design, Setting, and Subjects

This was a retrospective chart review of the electronic medical records of patients who have previously undergone gastrectomy with oncologic intent at the Hillel Yaffe Medical Center in Hadera, during the years of 2007–2017. The medical records were examined for a number of factors. Demographic data such as age and sex were noted, as well as history of previous abdominal surgery to identify sub-groups that may produce confounding results such as adhesions. Gastrectomies were classified as either total, subtotal, or proximal. Surgical and pathology records were reviewed to determine the T, N, and M staging of the treated gastric cancer. The pathology records were investigated to determine number of lymph nodes that were examined and the presence of lymphovascular invasion (LVI). This study was approved by the Institutional Review Board (protocol HYMC-17-104).

### Data Analysis

Adequate number of lymph nodes resected (15 or above) was defined as the dependent variable. Clinical risk factors for inadequate lymph node resection were assessed using the Fisher exact probability test, or the chi-square test for independence for categorical variables (sex, neoadjuvant therapy, previous surgery, operation, operative intent, T stage, N stage, M stage, and presence of lymphovascular invasion). Difference in median age between patients with adequate number of resected lymph nodes and patients with inadequate number of resected lymph nodes was analyzed using the Mann–Whitney test. Adequacy of lymph node resections per individual surgeon was assessed by linear regression. The number of operations done by each surgeon was compared to the percent of operations with adequate lymph nodes. Due to the small number of proximal gastrectomies in this cohort, these were considered together with subtotal gastrectomies when assessing the association of the extent of surgery (subtotal/proximal versus total) on the adequacy of lymph node resection. The combined effect of variables that may be associated with adequacy of lymph node resection was investigated using logistic regression. Since information concerning LVI was missing in a significant number of patients, two logistic regression models were created to investigate the combined effect of variables that may be significantly related to inadequate number of lymph nodes resected in the gastrectomy specimen: Model 1, without LVI; Model 2, with LVI. Variables with *P*-value threshold of ≤0.25 in the univariate analysis were included in both models as indicated by Hosmer and co-workers.^11^ Possible interactions between the variables were incorporated in each one. Only subjects with complete data were included in each analysis.^12^ Results are presented as odds ratio and 95% confidence intervals. Data were analyzed using dedicated statistical programs (GraphPad Instat 3.06 and GraphPad Prism 6.00 versions for Windows, GraphPad Software Inc., San Diego, CA, USA; SPSS Statistics for Windows, version 25.0, IBM Corp., Armonk, NY, USA). *P*-values less than 0.05 were considered significant. Numbers, percentages, and interquartile ranges (IQR) were approximated to the nearest tenth, and *P* and 95% confidence interval (95% CI) values to the nearest thousandth.

## RESULTS

Electronic medical files were screened, and patients undergoing ICD-9 coded procedures 43.5 to 43.99 between 2007 and 2017 were evaluated for possible inclusion in this analysis; 223 files were evaluated. Of these, 119 were excluded for the following reasons: 74 underwent gastric resection for morbid obesity (wrongly coded); 23 underwent gastric resection for peptic ulcer complication; five operations were for trauma; five gastric resections were done for tumors other than gastric cancer; five underwent wedge resections for either gastrointestinal stromal tumors (GIST) or other benign pathology; four patients underwent gastric resection for a benign pathology, and lymph nodes were not counted (duplication cyst, ectopic pancreas, benign GIST, severe mucosal hyperplasia); one patient was operated for gastric remnant cancer (following a previous gastric resection); one patient was operated for gastrostomy complication; and one patient was operated for removal of a foreign body.

A total of 104 patients were included in this study. All these patients had undergone formal gastric resection with oncologic intent, and the number of lymph nodes excised during gastrectomy was available for evaluation. Median age was 73 years (range 34 to 92); 43 patients were female and 61 were male. Neoadjuvant therapy was performed in only three patients. Previous history revealed 18 had undergone previous abdominal surgery, and 84 had not; in 2 patients, details concerning previous surgery were missing. Twenty patients underwent total gastrectomy, 71 patients underwent subtotal gastrectomy, and 13 patients underwent proximal gastrectomy. Surgery was performed with curative intent in 91 patients and with palliative intent in 13 patients. The proportion of patients with adequate number of lymph nodes assessed (15 and over) was 49.0% (51/104). Thirty-day mortality, sixty-day mortality, and ninety-day mortality were 2.9%, 3.8%, and 6.7% respectively.

**Figure 2:**
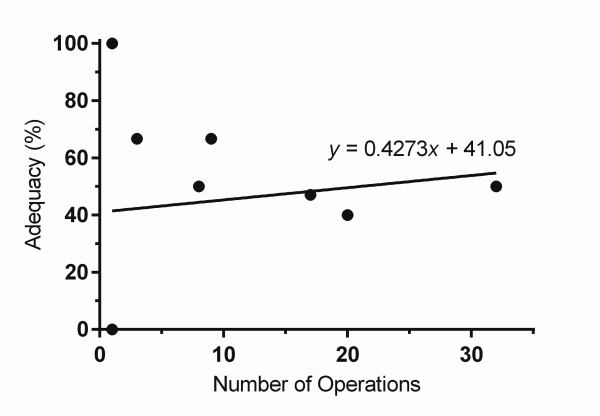
Adequacy as a Function of Surgical Experience Percent of operations with adequate number of lymph nodes resected as a function of number of surgeries done by individual surgeons.

Table 1 presents univariate analysis exploring possible risk factors for inadequate number of lymph nodes assessed. In two patients, information concerning previous abdominal surgery could not be clarified. Seven patients had malignancies other than adenocarcinoma, accounting for missing data on T stage in these patients. In one patient with diffuse metastatic disease, T stage could not be defined by the pathology report. In 18 patients LVI was not reported in their pathology report. Both “extent of operation less than total gastrectomy” and “no LVI” were significant factors in univariate analysis predicting inadequate lymph node resection. Increased relative risk for early T stage and no lymph node involvement with tumor did not reach clinical significance. Figure 2 presents the association of number of surgeries done by any single surgeon and the percent of operations with adequate number of lymph nodes resected. No association was found between the number of surgeries done by individual surgeons and their success in resecting an adequate number of lymph nodes (*P*=0.678).

**Table 1:** Univariate Analysis of Possible Risk Factors for Inadequate Number of Lymph Nodes Assessed in Patients Undergoing Gastrectomy with Oncologic Intent.

Variable	Number of Lymph Nodes Assessed		Relative Risk (95% Cl)	*P* Value

	Inadequate: Patients, *n* (%)	Adequate: Patients, *n* (%)		
**Sex**			1.088 (0.745, 1.587)	0.695
Female	23 (53.5)	20 (46.5)		
Male	30 (49.2)	31 (50.8)		

**Median Age in Years [range]**	74 [42–92]	71 [34–92]	--	0.409

**Neoadjuvant Therapy**			0.647 (0.129, 3.245)	0.614
Treatment	1 (33.3)	2 (66.6)		
No Treatment	52 (51.5)	49 (48.5)		

Previous Surgery*			1.436 (0.963, 2.141)	0.193
Yes	12 (66.7)	6 (33.3)		
No	39 (46.4)	45 (53.6)		

**Gastrectomy**			1.865 (0.93, 3.741)	0.047
Subtotal/Proximal	47 (56.0)	37 (44.0)		
Total	6 (30)	14 (70)		

**Intent**			0.894 (0.481, 1.661)	0.773
Palliative	6 (46.2)	7 (53.8)		
Curative	47 (51.6)	44 (48.4)		

T Stage*			1.542 (1.042, 2.281)	0.053
HGD-T2	21 (63.6)	12 (36.4)		
T3–T4	26 (41.3)	37 (58.7)		

**N Stage**			1.468 (1.009, 2.136)	0.051
N0	28 (62.2)	17 (37.8)		
N1–3	25 (42.4)	34 (57.6)		

**M Stage**			1.119 (0.602, 2.080)	0.773
M0	47 (51.6)	44 (48.4)		
M1	6 (46.1)	7 (53.8)		

Lymphovascular Invasion*			1.774 (0.364, 0.872)	0.014
No	20 (64.5)	11 (35.5)		
Yes	20 (36.4)	35 (63.6)		

* Missing data: previous surgery, *n=*2; T stage, *n=*8; lymphovascular invasion, *n=*18.

CI, confidence interval; HGD, high-grade dysplasia.

For Model 1, the combined effects of operation type (total versus subtotal/proximal), previous surgery, T stage, and N stage were investigated using logistic regression (Table 2), while the same variables and LVI were investigated in Model 2 (Table 3). For Model 1, there were no significant results when all variables were analyzed together. Possible interaction effects between the variables were also all insignificant. Analysis of all variables together in Model 2 revealed that partial gastrectomy, either proximal or subtotal, was the only independent predictor of inadequate lymph node resection.

**Table 2:** Logistic Regression Model 1 without Lymphovascular Invasion (94 Operations with Complete Data).

Variable	Odds Ratio	95% Confidence Interval	*P* Value
Subtotal/proximal gastrectomy	0.456	0.149, 1.391	0.168
Previous abdominal surgery	0.324	0.100, 1.048	0.060
T stage	4.285	0.989, 18.568	0.052
N stage	1.700	0.355, 8.144	0.507

**Table 3:** Logistic Regression Model 2 with Lymphovascular Invasion (82 Operations with Complete Data).

Variable	Odds Ratio	95% Confidence Interval	*P* value
Subtotal/proximal gastrectomy	0.253	0.067, 0.958	0.043
Previous abdominal surgery	0.318	0.089, 1.138	0.078
T stage	2.913	0.504, 16.843	0.232
N stage	1.066	0.177, 6.436	0.944
Lymphovascular invasion	2.192	0.544, 8.832	0.270

## DISCUSSION

Gastric cancer is the fourteenth most common form of cancer in Israel, but the ninth leading cancer directly causing mortality.^13^,^14^ In 2013, 745 new cases were diagnosed in Israel, including both Jewish and Arab, male and female subpopulations.

The retrieval of a greater number of lymph nodes in a gastrectomy for gastric cancer has previously been found to have a positive impact on patient survival rates.^15^ In the present study, the proportion of patients in whom surgeons successfully retrieved at least 15 lymph nodes was 49.0%. Previous studies have shown proportions that vary widely, ranging from adequate retrieval rates of 87% to only 21%.^16^,^17^ Mirkin et al. published a study relying on the National Cancer Data Base, which captures over 70% of the newly diagnosed cancer patients in the United States.^10^ In their study, as many as 60% of the patients undergoing gastrectomy for gastric cancer had 15 lymph nodes or less examined by the pathologist.

Successful retrieval of adequate number of lymph nodes is less commonly reported in studies taking place in the Western Hemisphere, whereas adequate lymph node retrieval occurs more frequently in the East. This may be due to higher rates of gastric cancer incidence in countries such as Japan, leading to greater awareness by physicians and more aggressive gastrectomies.^18^,^19^ Growing gastric cancer rates and awareness of improved results in Japanese studies may be leading to the increase in lymph node retrieval in Western countries such as the United States.^20^

Although there is ample evidence that suggests that the retrieval of 15 lymph nodes or more improves survival among gastric cancer patients undergoing gastrectomy, there has been little investigation regarding which, if any, real-world factors may influence the lymph node retrieval. One previous study by Coburn et al. found the following factors which were associated with adequate lymph node retrieval: higher stage, worse grade, age <74 years, later year of diagnosis, non-white race, more extensive surgery, female sex, and “SEER region” (region in the US where the surgery was performed).^17^ Unlike Coburn et al. we found significant association between neither the age nor sex and the successful retrieval of at least 15 lymph nodes. Gastric cancer is a disease primarily affecting older individuals with a positive skew for males; our results seemingly indicate that the surgical approach and outcome were similar, regardless of age or sex. Our results are also in disagreement with Coburn et al. regarding the significance of the staging of the cancer in affecting adequate lymph node retrieval. According to Coburn et al, tumor factors are important in the successful retrieval of a larger number of lymph nodes, whether the lymph nodes are involved with tumor or not involved. T and N tumor staging in this study were almost significant when examined using univariate analysis, but the association became insignificant when examined with logistic regression analysis.

Gastrectomies involve the resection of an extensive amount of gastric and perigastric tissue, more or less tissue depending on the type of gastrectomy. In our study 20 patients underwent total gastrectomy, whereas 84 patients underwent subtotal or proximal gastrectomy. Similar to the results in Coburn et al., we found a significant positive relationship between the extent of the gastric resection and adequate number of lymph nodes retrieved.^17^

## LIMITATIONS

Certain limitations should be taken into consideration. Only 104 patients were included in this study. Increasing the sample size might have detected a significant association between decreased T stage and N stage and inadequate lymphadenectomy. Still, it was our objective to evaluate major associations. Furthermore, as Hosmer and co-workers advocated, variables with *P*-value threshold of ≤0.25 in the univariate analysis were included in both logistic regression models.^11^ Differences observed for T and N stages remained non-significant.

We did not include the surgeon’s intention to perform a D1 or D2 lymph node resection as an independent variable in this study. As described in the introduction, lymphadenectomy’s nomenclature may be surgeon-related. Berlth et al. reported on 460 patients undergoing gastrectomy for gastric adenocarcinoma.^21^ Though the extent of lymph node resection was defined as D2 in these patients, the number of lymph nodes assessed was variable, ranging from 10 to 91.

## CONCLUSION

In this study, fewer than 15 lymph nodes were available for pathological evaluation in about half of the patients undergoing gastrectomy with oncologic intent. The extent of resection was the only factor significantly associated with removing at least 15 lymph nodes. Patient factors (age and sex) and tumor factors (TNM staging, LVI) had no bearing on adequate lymph node retrieval. The volume of previous surgeries performed by the surgeon was also found to not have a relationship with adequate lymph node number retrieval.

## Abbreviations

LVI: lymphovascular invasion.
